# Flubendazole Elicits Antitumor Effects by Inhibiting STAT3 and Activating Autophagy in Non-small Cell Lung Cancer

**DOI:** 10.3389/fcell.2021.680600

**Published:** 2021-08-26

**Authors:** Xiaona Xie, Xueding Cai, Yemeng Tang, Chunhui Jiang, Feng Zhou, Lehe Yang, Zhiguo Liu, Liangxing Wang, Haiyang Zhao, Chengguang Zhao, Xiaoying Huang

**Affiliations:** ^1^The First Affiliated Hospital, Wenzhou Medical University, Wenzhou, China; ^2^School of Pharmaceutical Sciences, Wenzhou Medical University, Wenzhou, China; ^3^The Institute of Life Sciences, Wenzhou University, Wenzhou, China

**Keywords:** flubendazole, NSCLC, antitumor, autophagy, STAT3

## Abstract

Non-small cell lung carcinoma (NSCLC) is a major neoplastic disease with a high mortality worldwide; however, effective treatment of this disease remains a challenge. Flubendazole, a traditional anthelmintic drug, possesses potent antitumor properties; however, the detailed molecular mechanism of flubendazole activity in NSCLC needs to be further explored. In the present study, flubendazole was found to exhibit valid antitumor activity *in vitro* as well as *in vivo*. Flubendazole blocked phosphorylation of STAT3 in a dose- and time-dependent manner and regulated the transcription of STAT3 target genes encoding apoptotic proteins. Further, flubendazole inhibited STAT3 activation by inhibiting its phosphorylation and nuclear localization induced by interleukin-6 (IL-6). Notably, the autophagic flux of NSCLC cell lines was increased after flubendazole treatment. Furthermore, flubendazole downregulated the expression of BCL2, P62, and phosphorylated-mTOR, but it upregulated LC3-I/II and Beclin-1 expression, which are the main genes associated with autophagy. Collectively, these data contribute to elucidating the efficacy of flubendazole as an anticancer drug, demonstrating its potential as a therapeutic agent via its suppression of STAT3 activity and the activation of autophagy in NSCLC.

## Introduction

Non-small cell lung carcinoma (NSCLC) refers to the widest variety of carcinomas and primarily causes carcinoma-associated deaths globally, as suggested by the latest annual report on global carcinoma statistics (Hua et al., 2020). Although early NSCLC diagnosis processes and treatment have advanced with the use of novel techniques and targeted therapies, the 5-year total survival of patients remains low (15%) (Keith and Miller, 2013; Cortes-Dericks and Galetta, 2019). Patients with advanced NSCLC experience poor prognosis, and a successful therapeutic intervention has yet to be identified. In addition, details regarding the initiation and progression of NSCLC need further elucidation. Therefore, the mechanisms underlying NSCLC development need to be identified to develop new and highly efficient therapies targeting the molecular level.

In tumorigenesis, the signal and activator of transcription 3 (STAT3) signaling pathway orchestrates a wide range of biologically related processes, including uncontrolled cell proliferation, anti-apoptosis, migration, and angiogenesis (Yu et al., 2014; Banerjee and Resat, 2016). Moreover, STAT3 is constitutively active in various carcinomas, including colon, prostate, breast, and lung carcinomas (Mora et al., 2002; Banerjee and Resat, 2016; Huynh et al., 2019; Lin et al., 2019; Wei et al., 2019; Yang et al., 2020; Zhao et al., 2020). Indeed, inhibiting STAT3 activation represents a targeted therapeutic strategy to treat carcinomas (Huynh et al., 2019; Wei et al., 2019). Various tools and resources have been applied for in-depth studies on the mechanisms underlying carcinoma progression, and STAT3 activity has emerged as an important mechanism for regulating carcinogenesis and metastasis (Yang et al., 2020; Zhao et al., 2020). Thus, the development of STAT3 inhibitors continues to be an active area of research, since no other effective inhibitory agents have been approved for treating NSCLC.

Autophagy refers to an autoregulatory process where double-membrane autophagosomes sequester aging or malfunctioning organelles and damaged proteins and then degrade them in autolysosomes, allowing cells to maintain their cellular homeostasis (Dikic and Elazar, 2018; Doherty and Baehrecke, 2018). Recent studies have shown that autophagy exerts many pathophysiologically related effects on numerous diseases, including carcinoma, heart disease, aging, autoimmune diseases, and neurodegeneration. Autophagy helps cells to clear the damaged proteins, and it is considered a programmed cell death type II mechanism, a type of cell death regulatory process (Edinger and Thompson, 2004; Mizushima, 2005; Kundu and Thompson, 2008; Mizushima et al., 2008; Yang and Klionsky, 2010). Moreover, autophagy critically impacts tumorigenesis, and growing evidence has indicated that a considerable number of therapeutic strategies trigger cell apoptosis by upregulating autophagy (Zheng et al., 2019). Nevertheless, the specific molecular-related mechanisms linking autophagy and apoptosis have yet to be ascertained. Therefore, an analysis of the autophagy-stimulating process is required to elucidate the underlying autophagy-mediated antitumor effect (Li et al., 2020). Thus, a study on whether autophagy activation provides an alternative cell death mechanism that can be used as a single therapeutic target for carcinoma therapy is significant.

Flubendazole is a member of the benzimidazole derivative drug class and has been shown to be effective in several countries for human treatment of gastrointestinal nematodes (Mackenzie and Geary, 2011). Notably, flubendazole also exhibits anticarcinoma activity in a wide range of carcinomas, including breast carcinoma, melanoma, colorectal carcinoma, and leukemia (Spagnuolo et al., 2010; Čáňová et al., 2018; Hanušová et al., 2018; Kim et al., 2018). Emerging preclinical evidence indicates that flubendazole is a promising anticarcinoma drug that is capable of inhibiting tubulin polymerization processes, thereby inducing a mitotic catastrophe (Spagnuolo et al., 2010; Kim et al., 2018). Nevertheless, the antitumor mechanisms of flubendazole in NSCLC have not been fully illustrated. In the present study, our aim was to characterize the mechanisms of flubendazole activity in NSCLC and its association with the inhibition of the STAT3 signaling pathway and stimulation of autophagy *in vitro* and *in vivo*.

## Materials and Methods

### Cell Lines and Culture

Human lung cancer cell lines H460, A549, and PC-9; the human bronchial epithelial cells BEAS-2b; and human umbilical vein endothelial cells (HUVEC) were purchased from the Cell Resources Center of the Shanghai Institutes of Biological Sciences (Chinese Academy of Sciences, Shanghai, China). H460 and A549 cell lines were maintained in RPMI 1640 culture medium including 10% fetal bovine serum (FBS). PC-9 cells were cultured in DMEM supplemented by 10% FBS. Human bronchial epithelial cells BEAS-2b and HUVECs were cultured in RPMI 1640 medium supplemented by 15% FBS. All cell lines were incubated at 37°C in a 5% CO_2_ atmosphere.

### Antibodies and Reagents

All cell culture reagents used in the study were purchased from Invitrogen Life Technologies (Carlsbad, CA, United States). The following antibodies were purchased from Cell Signaling Technology (Danvers, MA, United States): anti-STAT3 (1:1,000), anti-GAPDH (1:1,000), anti-MCL1 (1:1,000), anti-phosphorylated (p)-mTOR (Ser 2448) (1:1,000), anti-mTOR (1:1,000), anti-LC3-I/II (1:1,000), and anti-Beclin-1 (1:1,000). The following antibodies were purchased from Abcam (Cambridge, MA, United States): anti-p-STAT3 (p-STAT3) (1:4,000), anti-VEGF (1:4,000), anti-Lamin B1 (1:4,000), and anti-BAX (1:4,000). The BCL2 antibody and secondary antibodies horseradish peroxidase (HRP)-conjugated donkey anti-rabbit IgG and HRP-conjugated goat anti-mouse IgG were acquired from Santa Cruz Biotechnology Inc. (Dallas, TX, United States). Sigma-Aldrich (St. Louis, MO, United States) provided methylthiazolyldiphenyl-tetrazolium bromide (MTT) and dimethyl sulfoxide (DMSO). Bio-Rad Laboratories provided the polyvinylidene fluoride (PVDF) membrane and the chemiluminescence reagents and a Bradford protein test reagent. GenePharma (Shanghai, China) provided small interfering RNAs (siRNAs) targeting STAT3 (si-STAT3) and the respective negative control siRNA (si-NC). Invitrogen Life Technologies (Carlsbad, CA, United States) provided transfection liposome Lipofectamine 3000. BD Pharmingen (Franklin Lakes, NJ, United States) provided the Annexin V-fluorescein isothiocyanate (FITC) Apoptosis Detection Kit I and propidium iodide (PI). Tandem monomeric RFP-GFP-tagged LC3 was purchased from Gene Pharma (Shanghai, China). Flubendazole (HPLC ≥ 98%) was supplied by Sigma-Aldrich (St. Louis, MO, United States). Bafilomycin A1 (HY-100558) was purchased from MedChemExpress (MCE, NJ, United States).

### MTT Assay

Viability and cytotoxicity of human NSCLC cells and normal human cells were measured using MTT. Next, flubendazole at different concentrations was added to the cells for 48 h, and subsequently, the MTT solution was added. After 4 h, the formed crystals were dissolved in 150 μl DMSO and were then analyzed using a microplate reader at 490 nm. Growth suppression was ultimately measured using the MTT assay, and growth curves were generated using GraphPad Prism 7.0 (GraphPad Software Inc., La Jolla, CA, United States), and the half-maximal inhibitory concentrations (IC50s) were calculated.

### Colony Formation Assay

Cells were seeded into a six-well plate at a concentration of 500 cells/ml. The cells were incubated at 37°C in a 5% CO_2_ atmosphere. On day 2, DMSO (control) and flubendazole at different concentrations (0, 0.5, 1, and 2 μM) were added, and the cells were incubated for an additional 24 h. The culture medium was removed, and a fresh culture medium without the treatment was applied within 2 days to support cell growth for 1 week. Colonies were washed with PBS prior to the staining protocol by using 4% paraformaldehyde and were then stained using 0.1% crystal violet for 10 min at ambient temperature.

### Flow Cytometry

Flubendazole at different concentrations (0, 0.5, 1, and 2 μM) was added to H460 and PC-9 cell cultures for approximately 36 h. Annexin V-FITC staining was used to detect apoptotic cells to explore the variations within the cell membrane during apoptosis. After the treatment period, the cells were resuspended in 500 μl binding buffer according to the manufacturer’s instructions for the apoptosis kit in a dark environment. The treated cells (as described above) were subsequently incubated with fluorescein-labeled Annexin V and PI. All samples were acquired by a flow cytometer (BD Biosciences), and the data were analyzed using FlowJo software.

### Hoechst 33342 Staining

H460, A549, and PC-9 cells were seeded into six-well cell culture plates and incubated overnight. Flubendazole at different concentrations (0, 0.5, 1, and 2 μM) was added to six-well plates for 36 h. Fixation was then conducted with 4% paraformaldehyde for 15 min at ambient temperature; the cells were washed with PBS three times and then subjected to a 30-min staining incubation with 33342 solutions at 25°C. Under a fluorescence microscope with ×200 amplification, apoptotic characteristics of cell death were determined according to the cell nucleus morphology. Four microscopic fields were randomly selected for observation in each group. Apoptotic cells were defined as cells that had brighter and smaller nuclei than those of normal cells. Apoptotic cells were detected by fluorescence microscopy (Nikon, Tokyo, Japan), using appropriate filters for blue fluorescence.

### Wound Healing Assay

PC-9 cells were grown to 80–90% confluence in six-well plates and were allowed to adhere overnight. Then, using a sterile 10-μl pipette tip, a scratch was formed by wiping the sterile center of the cell monolayer in each well. Next, the cells were washed with PBS three times, and the subsequent culture process was performed under serum-free conditions to monitor cell migration. Flubendazole (0, 0.5, 1, and 2 μM) was added to cells, and the cells were observed after the addition at 0 and 24 h. The rate of mobility was quantified based on the migrated distance of the cells from the reference lines to the center, compared with the controls. All experimental processes were repeated three times.

### Western Blotting Analysis

Proteins of cells and tissues were extracted using a tissue or cell protein lysate buffer (Total Protein Extraction Kit). A549, PC-9, and H460 cells were seeded in a six-well plate at a density of 5 × 10^5^ cells per well. Subsequently, flubendazole (0, 0.5, 1, and 2 μM) was added into six-well plates. Briefly, proteins underwent a separation process using 10% or 12% sodium dodecyl sulfate-polyacrylamide gel (SDS-PAGE). They were subsequently transferred onto a PVDF membrane and then blocked for 1.5 h in 5% skim milk. The blots were incubated with a specific primary antibody overnight at 4°C, followed by incubation with secondary antibodies. This density of immunoreactive bands was evaluated using ImageJ software.

### Transient Transfection of siRNA

GenePharma (Shanghai, China) designed and synthesized the siRNAs targeting STAT3 (si-STAT3) and the relative negative control siRNA (si-NC). Transient cell transfections were conducted in accordance with the manufacturer’s guidelines. siRNA sequences targeted STAT3 siRNA STAT3-Homo-398 5′-CCACUUUGGUGUUUCAUAATT-3′; STAT3 siRNA STAT3-Homo-978 5′-GCAACAGAUUGCCUGCAUUTT-3′; and STAT3 siRNA STAT3-Homo-1070 5′-CCCGUCAACAAAUUAAGAATT-3′.

### Cytoplasmic and Nuclear Protein Extraction

To separate the nuclear and cytoplasmic proteins of PC-9 cells, we employed the NE-PER Nuclear and Cytoplasmic Extraction Kit (Thermo Fisher Scientific, Waltham, MA, United States). PC-9 cells were stimulated for 30 min with interleukin (IL)-6 following flubendazole at different concentrations (0, 0.5, 1, and 2 μM) for 24 h. The nuclear and cytoplasmic protein extraction kit was used to obtain the cell lysate according to the manufacturer’s protocol. The protein expression of cytoplasmic and nuclear fractions was detected by western blotting.

### Immunofluorescent Staining

Cells were grown in a six-well dish at 37°C in 5% CO_2_ overnight. Subsequently, flubendazole (1 and 2 μM) was added to the cells for 24 h. The cells were subsequently fixed with 4% paraformaldehyde before incubation in 1% Triton X-100 for 15 min and 1% BSA at ambient temperature for 1 h. The cells were incubated with the specific primary antibody against p-STAT3 (1:300 in 3% BSA) and anti-LC3 primary antibodies (1:200 in 3% BSA) overnight at 4°C. Next, in a dark environment, a PE-labeled secondary antibody (1:300 in 3% BSA) was incubated with cells for 1 h, and 4′,6-diamidino-2-phenylindole dihydrochloride (DAPI) was used to stain the nuclei for 5 min. Images were captured under fluorescence microscopy (Nikon C2, Tokyo, Japan).

### Tandem GFP/mRFP-LC3 Transfection

A tandem monomeric RFP-GFP-tagged LC3 lentivirus purchased from GeneChem (Shanghai, China) was used to detect autophagy flux. GFP/mRFP-LC3 is a lentivirus overexpressing LC3 with two fusion proteins. Cells were seeded into six-well culture plates at 2 × 10^4^ cells per well. After incubation in a complete medium at 37°C in 5% CO_2_ for 24 h, the cells were transfected with tandem GFP/mRFP-LC3 for 12 h, by the manufacturer’s protocol. The cells were transfected for 48 h and then were exposed to flubendazole (1 and 2 μM). The samples were subsequently examined under a fluorescence microscope (Nikon C2, Tokyo, Japan).

### Animal Model

All experimental procedures were conducted according to the guidelines and the protocols approved by the Animal Policy and Welfare Committee of Wenzhou Medical University. Female athymic BALB/c nude mice (aged 6–8 weeks) were fed and maintained at experimental facilities in Wenzhou Medical University. Specific to the NSCLC xenograft model, a total of 5 × 10^6^ A549 cells were suspended in an equal volume of PBS and Matrigel to 100-μl aliquots and were subcutaneously implanted into the hind flank of nude mice. When the tumor volumes reached ∼50 mm^3^, the mice were divided into three groups (six mice per group). There were no apparent differences in the average body weight and tumor volume following intraperitoneal injection of flubendazole (10 and 20 mg/kg) and napabucasin (20 mg/kg). Treatments were administered every other day. The STAT3 inhibitor napabucasin was used as the positive control. The tumor length (l) and width (w) were used to determine the tumor volume (*V* = 0.5 × l × w^2^) before the first injection. On day 14 and 2 h after the last flubendazole treatment process, all mice were euthanized. The tumors were excised and weighed, and tissue lysates were used for western blotting analysis.

### Hematoxylin-and-Eosin Staining

The hearts, livers, kidneys, and lungs of animals were fixed in 4% paraformaldehyde and embedded in paraffin. The paraffin tumor tissue sections (5 μm) were deparaffinized and rehydrated and then stained with eosin and hematoxylin. The images were captured using a light microscope.

### Statistical Analysis

All data are expressed as mean ± standard deviation of single experiments repeated at least three times. Statistical analysis was performed with GraphPad Prism 8.0 software. Comparisons between two groups were analyzed by Student’s *t*-test, and multiple comparisons were analyzed by one-way analysis of variance using Dunnett’s *post hoc* test. *P*-value < 0.05 indicated statistical significance.

## Results

### Flubendazole Inhibited the Viability and Migration of NSCLC Cells

**FIGURE 1:**
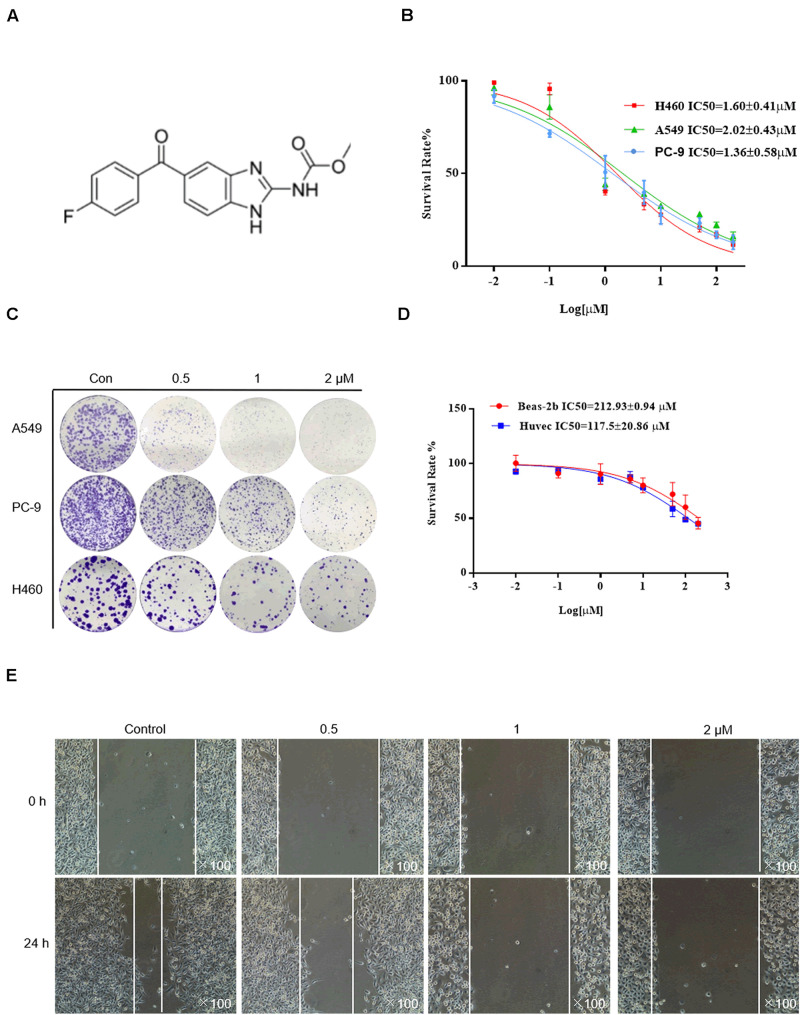
Flubendazole inhibited the viability and migration of lung cancer cells. **(A)** Chemical structure of flubendazole. **(B)** IC50 values of flubendazole in NSCLC cells. **(C)** Inhibitory impacts of flubendazole activity on cell colony formation. **(D)** IC50 values of flubendazole in HUVECs as well as common human bronchial epithelial cells BEAS-2b. **(E)** Representative figures of PC-9 cell migration assays. Images shown are representative of three separate experiments.

The chemical structure of flubendazole is presented in Figure 1A. To assess the inhibitory effect of flubendazole, cell viability was determined using the MTT assay. Flubendazole noticeably limited the viability of A549, H460, and PC-9 cells in a dose-dependent manner, with IC50 values of 2.02 ± 0.48, 1.60 ± 0.41, and 1.36 ± 0.58 μM, respectively (Figure 1B). Moreover, flubendazole significantly reduced colony formation in a dose-dependent manner (Figure 1C), which was consistent with the results of the viability assays. The inhibitory effects exerted by flubendazole in normal human cells BEAS-2b and HUVECs were significantly less compared to those exerted on carcinoma cells under the same exposure time, with an IC50 value at 48 h that is greater than 100 μM (Figure 1D). These data suggested that flubendazole effectively restricted the viability of NSCLC cells and that exposure to it was safe for normal cells. Moreover, the migration of PC-9 and A549 cells was reduced in a dose-dependent manner after treatment with flubendazole for 24 h (Figure 1E and Supplementary Figure 1A). Overall, treatment with flubendazole exerted a significant inhibitory effect on the viability and migration of NSCLC cell lines.

### Flubendazole Induced Apoptosis in Human NSCLC Cells

**FIGURE 2:**
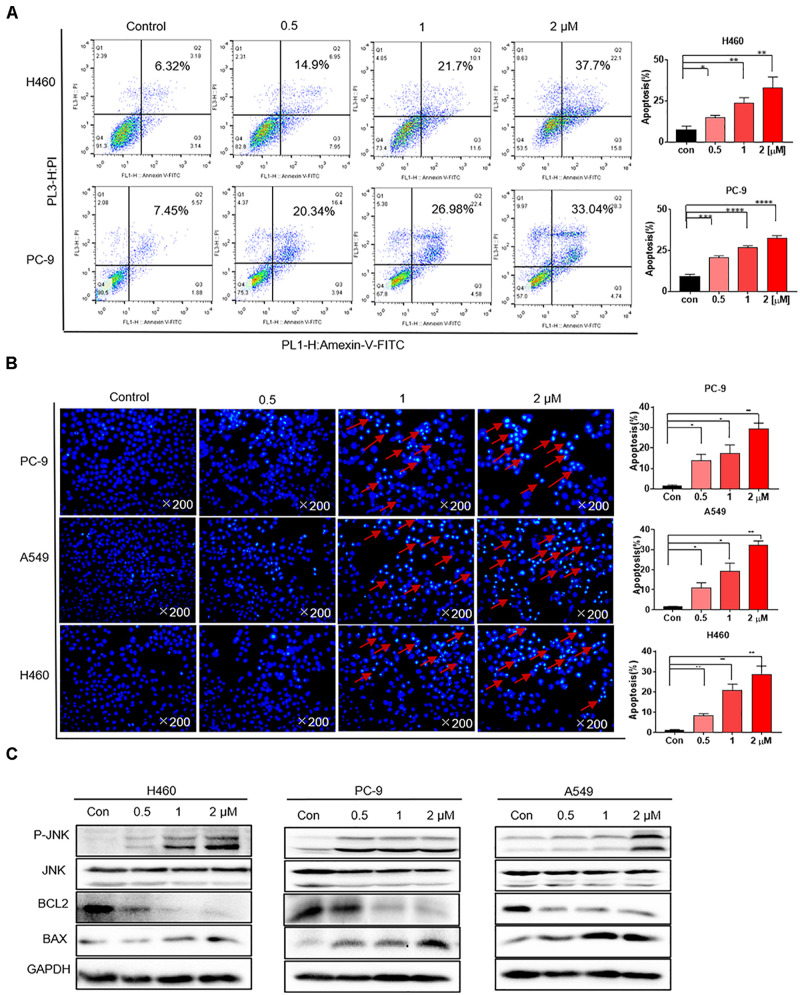
Flubendazole induced apoptosis in human lung cancer cells. **(A)** Cell apoptosis assays revealed the number of cells stained under positive Annexin V–PI when flubendazole treatment process was achieved. **(B)** Representative Hoechst 33342 staining shows the apoptotic morphology features pertaining to PC-9, H460, and A549 cells. Red arrows indicate apoptotic bodies. **(C)** Typical blots indicating the expression states of JNK, BCL-2, and BAX in human lung carcinoma cells. Images shown are representative of three separate experiments (*P < 0.05, **P < 0.01, ***P < 0.001, ****P < 0.0001).

H460 and PC-9 cells were treated with flubendazole at different concentrations (0, 0.5, 1, and 2 μM) for 36 h, followed by staining with Annexin V–FITC and PI to detect cell apoptosis. The proportion of apoptotic cells was evaluated using flow cytometry. Flubendazole markedly induced cell apoptosis in H460 and PC-9 cell lines in a dose-dependent manner (Figure 2A). Thereafter, cell nuclear morphology was observed using fluorescence microscopy. Apoptotic body formation was clearly identified in flubendazole-treated H460, A549, and PC-9 cells (Figure 2B). In addition, the observed apoptotic bodies increased with an increasing concentration of flubendazole by Hoechst staining. The apoptosis-associated protein expression of p-JNK and BAX was upregulated, whereas the expression of BCL2 was downregulated, further confirming the apoptotic effect of flubendazole (Figure 2C and Supplementary Figure 1B). Overall, the results indicated that flubendazole significantly promoted apoptosis in NSCLC cells.

### Flubendazole Inhibited STAT3 Signaling

**FIGURE 3:**
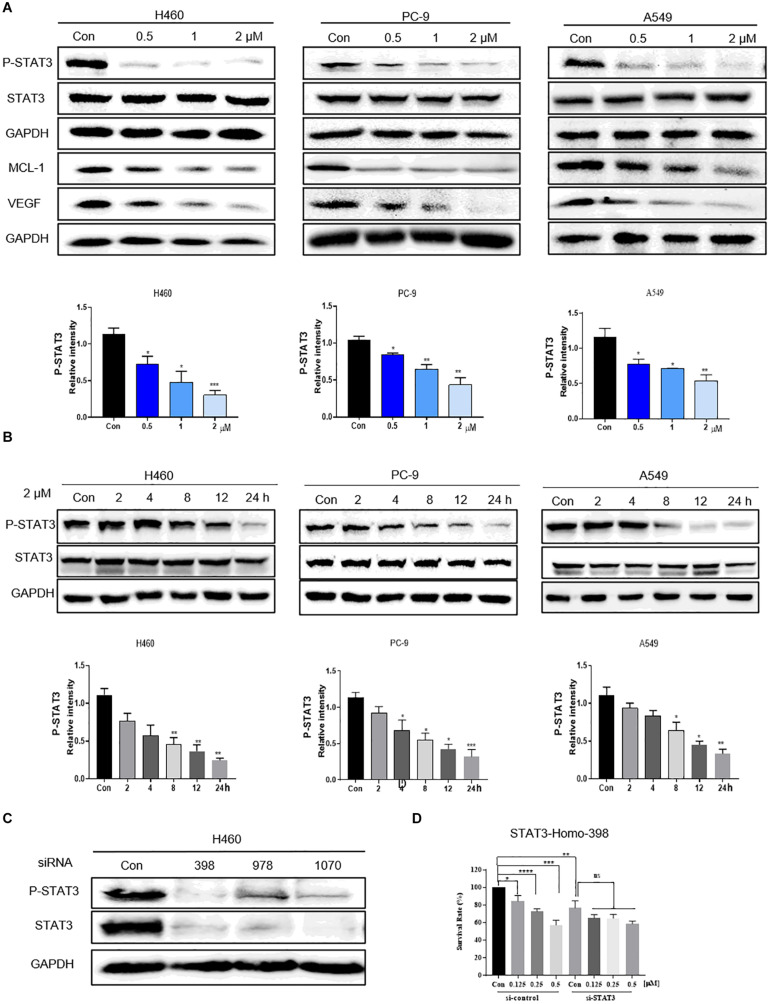
Flubendazole inhibited the STAT3 signaling pathway. **(A)** Expression levels of p-STAT3, STAT3, VEGF, and MCL-1 based on western blotting assays. **(B)** Flubendazole (2 μM) inhibited P-STAT3 in a time-dependent manner. **(C)** Expression of p-STAT3 and STAT3 detected by western blotting in H460 cells transfected using si-NC or si-STAT3 (si398, si978, and si1070) for 48 h. **(D)** Inhibitory effects of flubendazole on the proliferation of H460 cells after transfection with si-NC or si-STAT3 (si398). GAPDH as a loading control. Outcomes are representative of three independent experiments (*P < 0.05, **P < 0.01, ***P < 0.001, ****P < 0.0001).

Previous studies have mechanistically shown that flubendazole elicits antitumor effects by inhibiting STAT3 in triple-negative breast carcinoma and colorectal carcinoma (Oh et al., 2018; Lin et al., 2019). We thus assessed the expression of STAT3 by using flubendazole in NSCLC. Western blotting was used to determine the suppression of proteins involved in the STAT3 signaling pathway. The expression of p-STAT3 protein was markedly reduced by flubendazole treatment in NSCLC cells (Figure 3A). Treatment with flubendazole for 24 h lowered the expression state of the STAT3-associated protein VEGF and MCL-1 in PC-9, H460, and A549 cell lines in a dose-dependent manner. Under similar conditions, no apparent changes were observed in the STAT3 expression (Figure 3B). Whether the growth delay induced by flubendazole was correlated to the inactivation of STAT3 signaling was examined further. H460 cells were transfected transiently using three different si-STAT3 sequences (398, 978, and 1070) to knock down the expression of STAT3, with sequence 398 exhibiting the maximum inhibitory activity against STAT3 in H460 cell lines (Figure 3C). The results of the growth experiment demonstrated that flubendazole exerted significant effects the negative control siRNAs cells compared to siSTAT3, which were only slightly affected (Figure 3D). This finding suggested that flubendazole targeted STAT3 suppression in NSCLC cells.

### Flubendazole Inhibited Nuclear Translocation of STAT3

**FIGURE 4:**
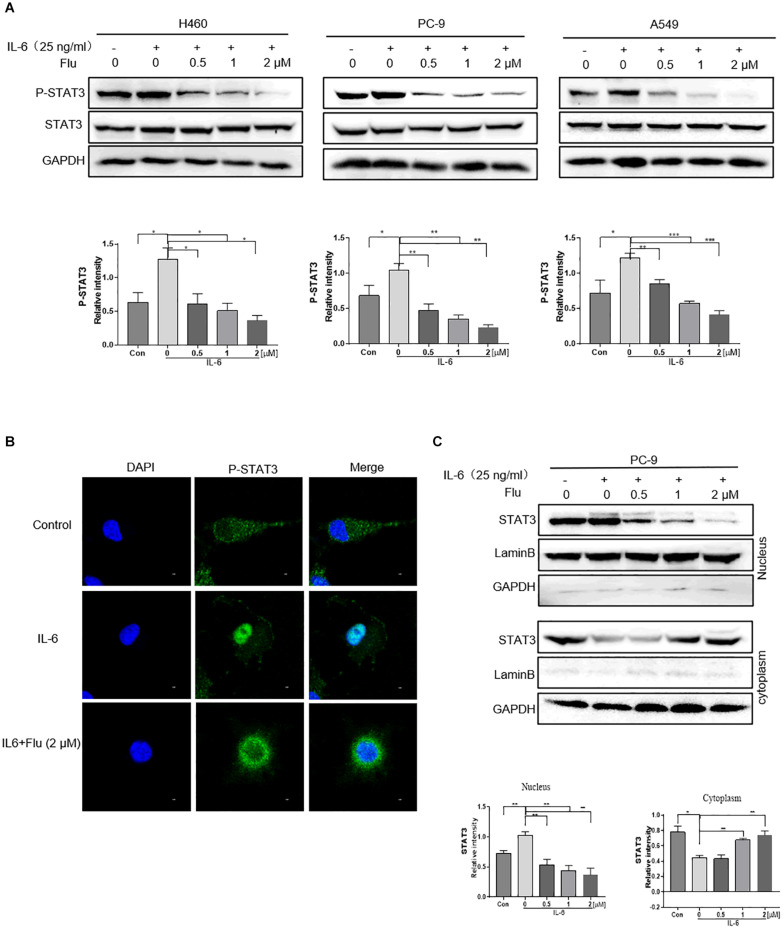
Flubendazole inhibited nuclear translocation of STAT3. **(A)** Expression of STAT3 in the cytosol and in the nucleus confirmed by western blotting assays. **(B)** Representative confocal microscopic images indicating the localization of p-STAT3 (green) and DAPI in PC-9 cells. **(C)** The expression levels of STAT3 in nuclear and cytosolic fractions were determined by using the western blot analysis. Images shown are representative of three separate experiments (*P < 0.05, **P < 0.01, ***P < 0.001).

We further investigated whether flubendazole treatment could influence the nuclear translocation of STAT3 in NSCLC cells. As shown in Figure 4A, stimulation with 25 ng/ml IL-6 for 30 min stimulated an increase in p-STAT3 levels. It is noteworthy that IL-6-triggered STAT3 activation could be blocked by pretreatment with flubendazole in the three NSCLC cell lines. Moreover, according to the immunofluorescence (IF) staining assay, IL-6 treatment resulted in a translocation of p-STAT3 from the cytoplasm to the nucleus, and this nuclear translocation could be inhibited by flubendazole in PC-9 cells (Figure 4B). Next, protein expression in cytoplasmic and nuclear extracts was subjected to western blotting, confirming that in cells exposed to flubendazole, the p-STAT3 nuclear translocation was significantly inhibited (Figure 4C). These results strongly confirmed the inhibitory effects of flubendazole on the IL-6-triggered nuclear translocation of p-STAT3 in NSCLC cells.

### Flubendazole Induced Autophagy in NSCLC Cells

**FIGURE 5:**
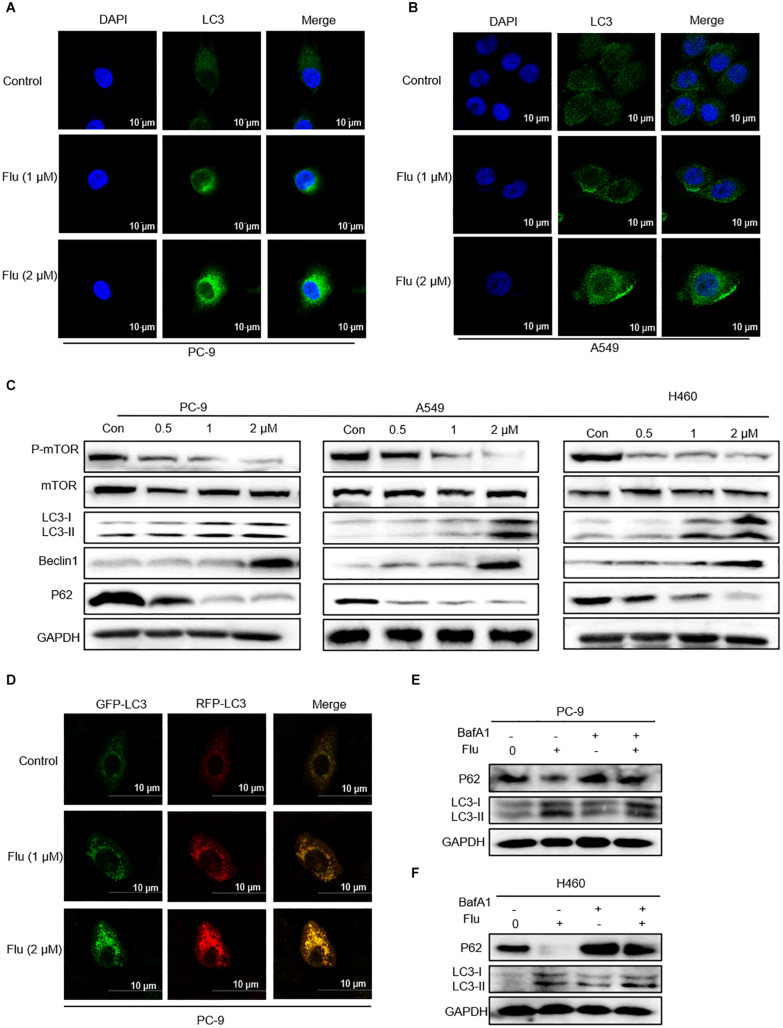
Flubendazole induced autophagy in NSCLC cells. **(A,B)** Immunofluorescence study of endogenous LC3 in PC-9 and A549 cells treated with flubendazole (1 and 2 μM) for 24 h. Representative figures with quantification of LC3 intensity. **(C)** Immunoblot analysis of p-mTOR, mTOR, p62, Beclin-1, and LC3 expression in three lung carcinoma cell lines treated with flubendazole (0.5, 1, and 2 μM) for 24 h. GAPDH as a loading control. **(D)** Different localization patterns of the GFP and RFP signals of the tandem fluorescent LC3′s (RFP-GFP-LC3). Cells transfected with plasmids that express tandem fluorescent LC3. Cells treated with 1 and 2 μM flubendazole at 24 h post-transfection and then analyzed by confocal microscopy. **(E,F)** PC-9 and A549 cells were co-incubated with flubendazole (1 μM) in the presence or absence of BafA1 (20 nM) for 24 h, and then the expressions of p62 and LC3 were detected. Results shown are representative of three independent experimental processes. Images shown are representative of three separate experiments.

Flubendazole is considered a potent inducer element of autophagy initiation and flux in human carcinoma treatment (Chauhan et al., 2015; Lin et al., 2019). Therefore, we verified whether flubendazole could cause autophagy in NSCLC cell lines. The LC3-II level is a hallmark of autophagy which correlates with the number of autophagosomes and/or autophagic degradation (Tanida et al., 2005). We detected a significant increase in the IF intensity of LC3 in A549 and PC-9 cells after flubendazole treatment (Figures 5A,B). Moreover, the classical autophagy markers mTOR and P62 were inhibited, while Beclin-1 and LC3-I/II expressions were upregulated by flubendazole (Figure 5C and Supplementary Figure 1C). To further identify the role of flubendazole in autophagy, PC-9 cells were first transfected with the GFP/mRFP-LC3 expression vector, and the formation of fluorescent autophagosomes (in green) and autolysosomes (in red) was further examined (Figure 5D). When an mRFP-GFP-LC3 construct is used, the GFP fluorescent autophagosomes signals can be seen, since the GFP signals are easily quenched in the acidic pH of autolysosomes. However, mRFP detects both autophagosomes and autolysosomes, as it is more stable under acidic conditions. The GFP signals (green puncta) indicate the initial process of autophagy, and the presence of mRFP (red puncta) signals is indicative of the later process of autophagy. Using this approach, we observed that the ratio of mRFP to GFP signals was significantly increased in flubendazole-stimulated PC-9 cells, indicating that the autophagic flux was enhanced without impeding autophagosome-lysosome fusion or autolysosome function (Figure 5D and Supplementary Figure 2C). Furthermore, unlike the control cells, when PC-9 cells are treated with flubendazole, a dose-dependent increase in yellow signals could be detected, indicating the presence of an autophagic body.

Bafilomycin A1 (BafA1), a lysosomal protease inhibitor that blocks autophagosome-lysosome fusion and LC3-II degradation, is a marker of the late process of autophagy. Upon BafA1 treatment, we observed a further increase in LC3-II signals in the cells. Zhou et al. also reported that co-treatment with BafA1 and flubendazole induced further accumulation of autophagosomes, suggesting that flubendazole-induced autophagy is a continuous process (Zhen et al., 2020). Similarly, we also detected increased accumulation of p62 and LC3 upon 24-h treatment with BafA1 (20 nM) and flubendazole (1 μM), suggesting that flubendazole may induce autophagic flux in NSCLC cells (Figure 5E and Supplementary Figures 2A,B). In addition, activation of JNK by flubendazole reduced the expression of BCL2 (Figure 2C). Further, Beclin-1 activity was inhibited by binding to BCL2. Thus, the suppression of BCL2 expression was a result of STAT3 blockade, and JNK activation released Beclin-1 from BCL2–Beclin 1 complexes, thereby triggering autophagy (Figures 2C, 5C). As shown in Figure 5C, NSCLC cell apoptosis was induced by autophagic initiation and the inhibition of BCL2 activity. Collectively, our findings indicated that flubendazole triggers apoptosis via autophagy stimulation in NSCLC cells.

### Flubendazole Inhibited the Growth of NSCLC Tumor Xenografts

**FIGURE 6:**
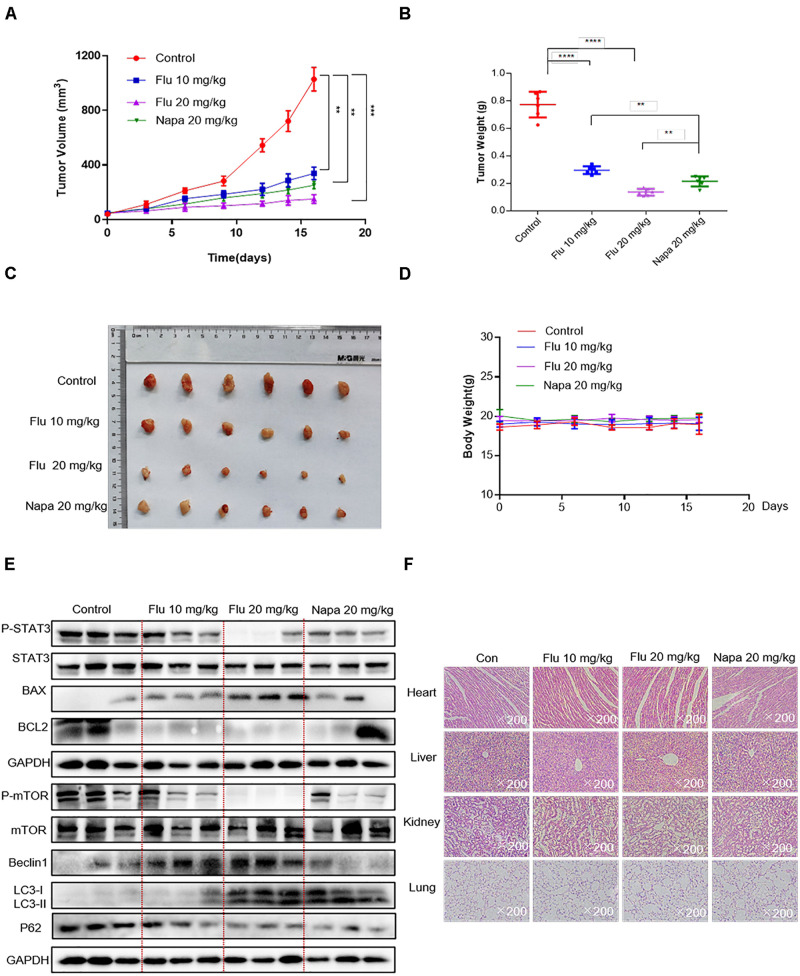
Flubendazole inhibited the growth of NSCLC tumor xenografts. **(A)** Tumor volumes. **(B)** Measurement of tumor weights. **(C)** Representative figures of the tumor tissue in the flubendazole-administered and control groups (*n* = 6). **(D)** Body weight of mice. **(E)** Representative blots indicating the expressions of p-STAT3, BAX, BCL-2 and p-mTOR, Beclin-1, and LC3 in tumor tissues. **(F)** Hematoxylin and eosin staining of the heart, liver, lung, and kidney tissues from nude mice at ×200 magnification. The images shown are representative of three separate experiments (**P < 0.01, ***P < 0.001, ****P < 0.0001).

Based on the convincing *in vitro* evidence, we next evaluated the potential therapeutic efficacy of flubendazole using human A549 xenograft models. The STAT3 inhibitor napabucasin was used as the positive control. BALB/c nude mice were treated with flubendazole (10 and 20 mg/kg) and napabucasin (20 mg/kg) every other day. Tumor volume and weight were notably decreased in the flubendazole-treated group as opposed to the vehicle group (Figures 6A–C). Moreover, the body weight of mice was stable in the flubendazole group (Figure 6D). We then investigated the effects of flubendazole on the *in vivo* tumor growth. Using protein lysates obtained from the xenografted tumor tissues, treatment with flubendazole caused a decrease in the levels of p-STAT3 and induced the expression of apoptosis protein of BAX; meanwhile, the BCL2 level was reduced. Simultaneously, the autophagic response exerted by flubendazole in the NSCLC *in vivo* model was also confirmed; the levels of Beclin-1 and LC3-I/II proteins increased, whereas those of mTOR and P62 were decreased following exposure to flubendazole (Figure 6E and Supplementary Figure 1D). In addition, flubendazole treatment did not induce any significant signs of gross toxicity in the heart, liver, kidney, or lung when compared to mice treated with vehicle alone (Figure 6F), thus demonstrating an excellent safety profile.

Altogether, these results indicated that flubendazole was a potential antitumor agent that could significantly inhibit tumor development by suppressing the STAT3 signaling pathway as well as by stimulating autophagy.

## Discussion

Flubendazole has found extensive application for the treatment of human infection against worms and intestinal parasites in recent decades (Mackenzie and Geary, 2011). As revealed by previous studies, flubendazole exhibits antitumor activity in various carcinomas, including colorectal carcinoma, myeloma, and triple-negative breast carcinoma both *in vitro* and *in vivo* (Čáňová et al., 2018; Kim et al., 2018; Lin et al., 2019), and is considered a promising antitumor agent (Kim et al., 2018; Lin et al., 2019). Nonetheless, the antitumor effects and mechanism of action of flubendazole in NSCLC have yet to be elucidated. Our findings revealed that flubendazole can markedly suppress cell proliferation and migration and could induce apoptosis *in vitro* (Figures 1, 2). Furthermore, the antitumor mechanism of flubendazole involves the inhibition of the STAT3 pathway. The present study also indicated that flubendazole could significantly suppress tumor development and did not induce any observable physiological toxicity in the xenograft NSCLC model. Flubendazole inhibits STAT3 activation and downstream STAT3 targeted gene expression, including VEGF, MCL-1, and BCL2. Meanwhile, flubendazole slightly influenced si-STAT3 cell activity, demonstrating the ability of flubendazole to reduce cell proliferation relying on STAT3. Furthermore, we showed that flubendazole could inhibit the IL-6-induced nuclear translocation of STAT3 phosphorylation. Altogether, these findings suggested that flubendazole could target STAT3 in human NSCLC.

Furthermore, we also examined the impact exerted by flubendazole on autophagic antitumor potency and verified its effect on human NSCLC cells. According to White et al., acute autophagy ablation in rats with NSCLC could block tumor growth and promote tumor cell death (Chen et al., 2013). Moreover, flubendazole exhibits anticarcinoma effects in triple-negative breast carcinoma by targeting EVA1A-modulated autophagy and apoptosis (Zhen et al., 2020). The role of the basal autophagy ratio in lung carcinoma requires further elucidation. Studies have indicated that flubendazole induces autophagic initiation by blocking mTOR activity and promoting lysosome–autolysosome maturation (Chauhan et al., 2015). Our findings showed that flubendazole could inhibit the classic autophagy markers mTOR and P62 as well as the upregulation of Beclin-1 and LC3-I/II. In addition, the immunofluorescent intensity of LC3 signals was significantly increased after flubendazole treatment (Figures 5A,B). In our study, we transfected human NSCLC cells with the GFP/mRFP-LC3 lentivirus to monitor autophagy and then treated them with flubendazole (1 and 2 μM). We then observed that signals indicating the presence of autophagic bodies increased in a dose-dependent manner relative to those in the control. These findings confirmed that flubendazole could potentially activate a complete autophagic flux in human NSCLC cells.

**FIGURE 7:**
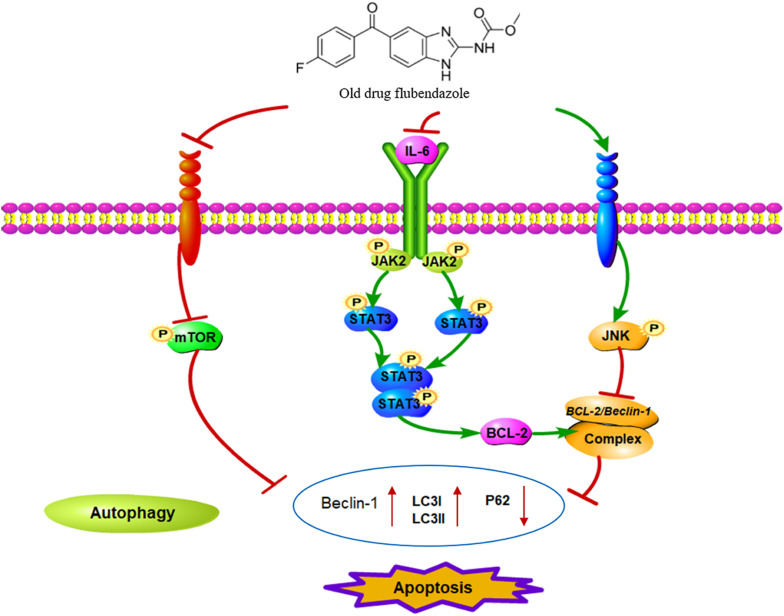
Schematic diagram of the potential antitumor mechanism of flubendazole via inhibition of STAT3 and activation of autophagy in cancer cells.

Flubendazole has recently been found to affect microtubule dynamics and to induce mitotic catastrophe and apoptosis (Chauhan et al., 2015; Čáňová et al., 2018; Lin et al., 2019). Treatment with flubendazole activates JNK-1, which in turn induces BCL2 phosphorylation (Chauhan et al., 2015), which results in the release of Bcl-2/Beclin-1 complex via microtubule acetylation. This flubendazole-induced autophagic flux may represent a rationale for the use of flubendazole for the treatment of diseases. Growing evidence has shown that STAT3 functions as the main transcription regulator of several genes associated with autophagy (Kang et al., 2012; Niso-Santano et al., 2013; Huang et al., 2014; Maycotte et al., 2014), including BCL2 and BECN1 (You et al., 2015; Sun et al., 2017). In the present study, we confirmed that flubendazole inhibited BCL2 expression and triggered cell apoptosis and autophagy (Figure 7). The above mechanisms provide a novel insight into the molecular activity of flubendazole as an effective anticarcinoma treatment. These findings demonstrate that apart from targeting STAT3, autophagy may represent effective flubendazole target against human NSCLC.

## Conclusion

In this study, flubendazole was described as an inhibitor of human NSCLC cell growth. Moreover, we also showed that flubendazole could mediate STAT3 downregulation, inhibit STAT3-mediated gene BCL2, and activate the autophagy system. Considering the safety profile of flubendazole and the associated preclinical studies, clinical trials should also consider the application of flubendazole for the treatment of carcinoma, particularly NSCLC. Collectively, our results shed light on the hypothesis that flubendazole can be further explored as a STAT3 inhibitor and an autophagy activator for the treatment of NSCLC. Subsequent studies are likely to confirm the roles of STAT3 and autophagy in tumor metastasis and chemoresistance and further investigate the synergistic impacts exerted by flubendazole with chemotherapies and targeted drugs.

## Data Availability Statement

The raw data supporting the conclusions of this article will be made available by the authors, without undue reservation.

## Ethics Statement

The study was conducted in accordance with the national guidelines and acquired the approval of the Ethics Committee of the First Affiliated Hospital of Wenzhou Medical University. All animal procedures were done according to the institutional laboratory animal research guidelines and were approved by the Wenzhou Medical University Animal Policy and Welfare Committee and were performed in accordance with the Declaration of Helsinki.

## Author Contributions

XX, XC, CJ, FZ, and LY carried out most of the experiments. XX, XC, YT, CJ, and FZ analyzed the data and prepared the figures. FZ, LY, ZL, LW, and HZ conceived the idea and designed the research. XX and XC wrote the manuscript. All authors read and approved the final version of the manuscript.

## Conflict of Interest

The authors declare that the research was conducted in the absence of any commercial or financial relationships that could be construed as a potential conflict of interest.

## Publisher’s Note

All claims expressed in this article are solely those of the authors and do not necessarily represent those of their affiliated organizations, or those of the publisher, the editors and the reviewers. Any product that may be evaluated in this article, or claim that may be made by its manufacturer, is not guaranteed or endorsed by the publisher.
