# Supplementary material for: Flubendazole Elicits Antitumor Effects by Inhibiting STAT3 and Activating Autophagy in Non-small Cell Lung Cancer
Source: Front Cell Dev Biol. 2021 Aug 26;9:680600. doi: 10.3389/fcell.2021.680600 (PMC8427440; doi:10.3389/fcell.2021.680600)
Supplement: Supplementary file 1 [file Data_Sheet_1.docx]

**Supplementary Material**

**Figure S1**


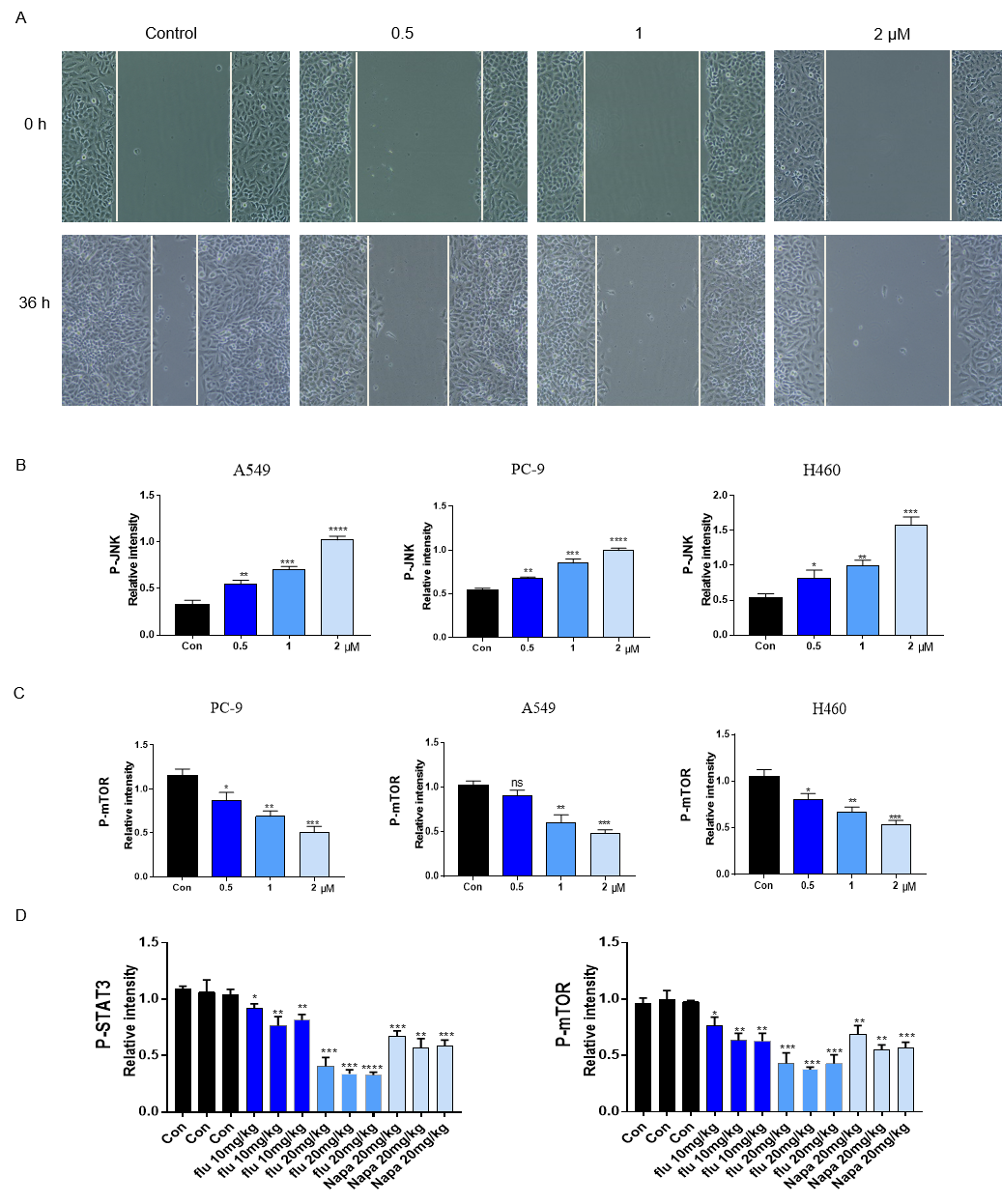


**Figure S2**


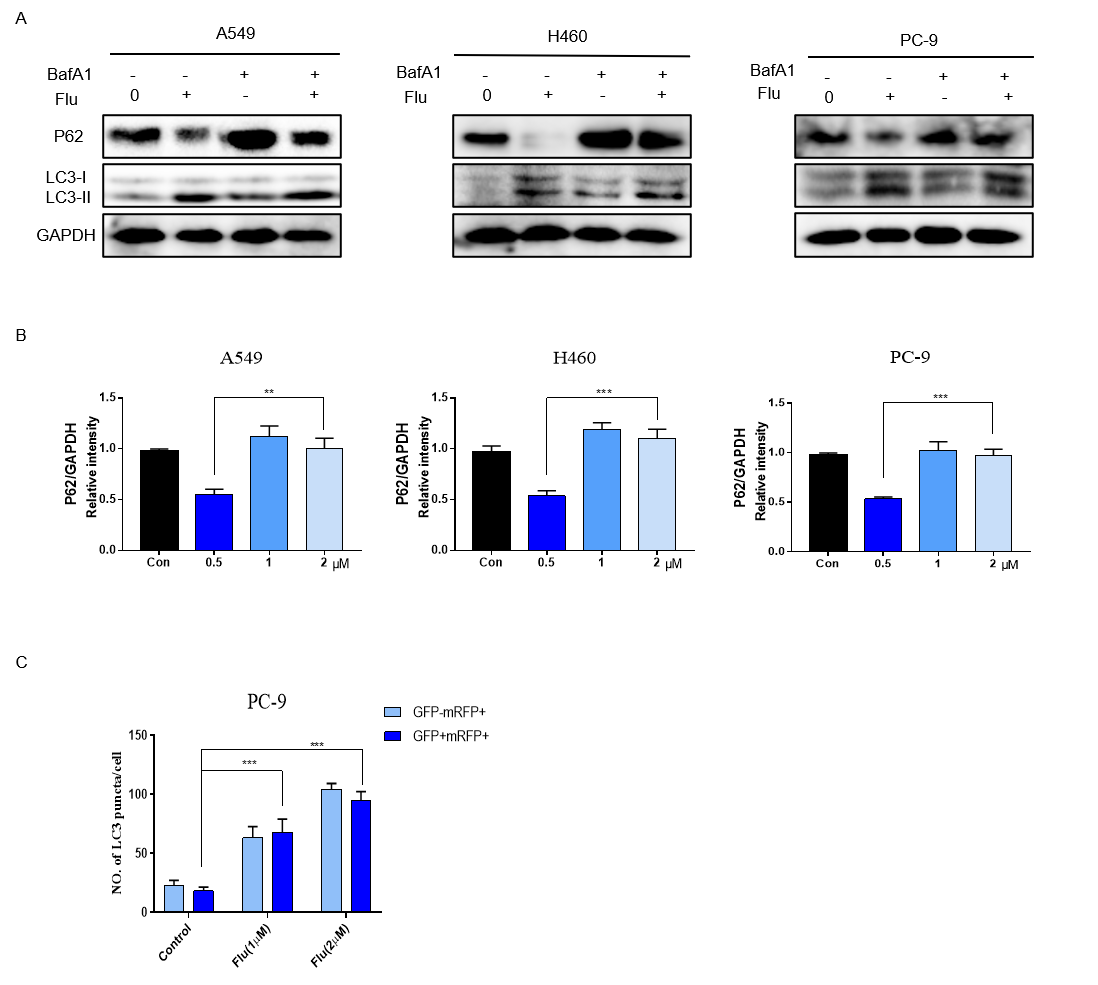


**Figure S1** (A) Representative figures of A549 cell migration assays. (B). Cells were treated with Flubendazole at different concentrations as indicated for 24 h, the cell lysates were processed for Western blot analysis for protein expression of p-JNK and the relative intensity was calculated. (C). Western blot analysis was used to detect the levels of p-mTOR after flubendazole treatment and the relative intensity was calculated. (D). Representative blots indicating the expression of p-STAT3, and p-mTOR in tumor tissues, and the relative intensity was calculated.

**Figure S2** (A) PC-9, H460 and A549 cells were co-incubated with flubendazole (1 µM) in the presence or absence of BafA1 (20 nM) for 24h, and then the expression of p62 and LC3 were detected. (B). Western blot analysis was used to detect the levels of p62 and LC3 after flubendazole treatment and the relative intensity was calculated. (C). The autophagosomes quantification analysis was calculated.
